# Pregnancy and delivery after laparoscopic mesh repair for postpartum diastasis recti abdominis: A case report

**DOI:** 10.1097/MD.0000000000043589

**Published:** 2025-08-01

**Authors:** Shota Eguchi, Yoshio Nagahisa, Kenji Yamaguchi, Michio Okabe, Toshihiko Masui

**Affiliations:** a Department of General Surgery, Kurashiki Central Hospital, Kurashiki City, Japan.

**Keywords:** diastasis recti abdominis, laparoscopic repair, pregnancy, vaginal delivery

## Abstract

**Rationale:**

Diastasis recti abdominis (DRA) refers to the separation of the 2 rectus abdominis muscles along the linea alba. It is a common condition during pregnancy and the puerperium that can cause musculoskeletal pain, urogynecologic symptoms, and body image concerns. However, surgery for this condition is controversial and there is no consensus on the indications for surgery. This case report aims to document a successful pregnancy and vaginal delivery following laparoscopic mesh repair, highlighting the potential safety and functional implications of surgical intervention in reproductive-age women.

**Patient concerns:**

A 29-year-old woman who had given birth vaginally 2 weeks before presented with bulging of the abdominal wall.

**Diagnoses:**

The patient was diagnosed with DRA.

**Interventions:**

A laparoscopic mesh repair was planned. The patient was informed preoperatively of the potential surgical risks associated with pregnancy and childbirth after the repair, including recurrence of the condition and restricted fetal growth. Polypropylene mesh (Ventralight Sepra Technology) was placed in the retroperitoneal space.

**Outcomes:**

Three years after the repair, the patient conceived naturally. Her pregnancy progressed without complications and she gave birth vaginally.

**Lessons:**

Placement of a mesh in the retroperitoneal space not only provides a good therapeutic outcome but may also allow pregnancy.

## 1. Introduction

Diastasis recti abdominis (DRA) is a condition in which the 2 rectus abdominis muscles separate to the left and right along the linea alba and is often triggered by pregnancy or childbirth.^[1]^ It is reported to occur in 1 in 3 women after childbirth.^[2]^ There is no standard treatment for DRA, and nor has a standardized surgical technique been established. We previously reported a case of DRA treated with a combination of an extended-view totally extraperitoneal approach, the Rives-Stoppa technique, and the transversus abdominis release.^[3]^ In this case report, the woman’s subsequent pregnancy and delivery are described.

## 2. Case report

A 29-year-old woman (weight: 45 kg, body mass index: 18.03 kg/m²), who had given birth vaginally 2 weeks before, presented with bulging of the abdominal wall. Her medical history was insignificant. Her pregnancy history was gravida 3 and para 2.

**Figure 1.**
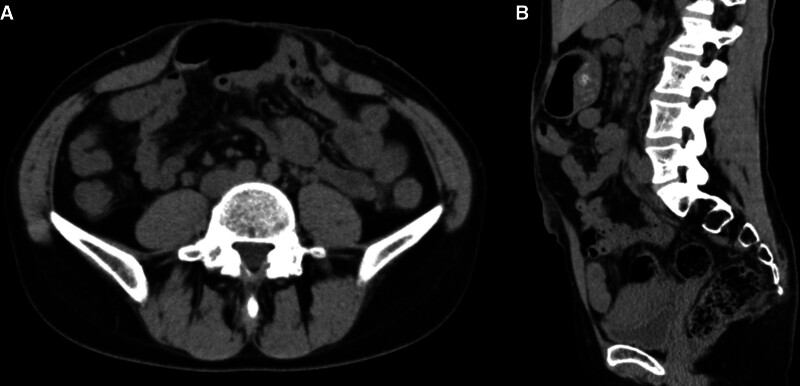
Preoperative computed tomography images of diastasis recti abdominis. Axial (A) and sagittal (B) views showing 6–7 cm of diastasis.

Computed tomographic and ultrasonographic examinations showed an attenuated linea alba and separation of the 2 rectus abdominis muscles (Fig. 1). The distance between the 2 rectus abdominis muscles was 7 cm at the umbilicus, and the separation continued to 4 cm above and 4 cm below the umbilicus. Three months of conservative therapy comprising exercise to strengthen the transversus abdominis muscle was ineffective. She also had lower abdominal colic pain, back pain, and difficulty in daily life, but imaging examinations ruled out other potential etiologies. From the perspective of cosmetic appearance and prevention of infection, bowel adhesion, and recurrence, we chose the enhanced-view totally extraperitoneal Rives-Stoppa technique and transversus abdominis release, nonabsorbable thread suture, and mesh reinforcement.

After induction of general anesthesia and endotracheal intubation, the patient was placed in the supine position. A 12-mm optical trocar was inserted in the right upper quadrant, and five 5-mm working ports were placed to access the retrorectus space bilaterally. After dissecting the posterior rectus sheath both cranially and caudally, bilateral transversus abdominis release was performed medial to the neurovascular bundles to develop a wide retromuscular plane. The rectus abdominis muscles were reapproximated using self-locking barbed 2-0 V-Loc™ sutures (Medtronic, Minneapolis). A large polypropylene mesh (30 × 21 cm, Ventralight Sepra Technology; Beckman, Dickinson and Company, Franklin Lakes) was placed in the retrorectus space and anchored using 3-0 Vicryl sutures to the cranial, caudal, and both lateral edges. A 19-French silicone suction drain was inserted into the retrorectus space. The postoperative course was uneventful; the drain was removed on postoperative day 4, and the patient was discharged on day 5.

**Figure 2.**
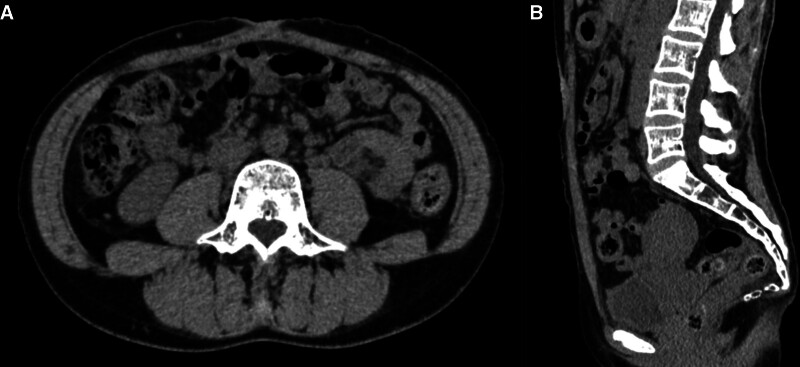
Computed tomography images 1 month after the delivery. Axial (A) and sagittal (B) views showing no recurrence of diastasis recti abdominis and no seroma.

One month after surgery, the woman had mild mood disorder while carrying her child, but her lower abdominal colic pain and back pain decreased day by day, and she had no pain from mesh fixation induced by contraction and relaxation of the rectus abdominis muscle. We explained the risk of chronic pain and recurrence of DRA during subsequent pregnancies. Three years after surgery, the woman was found to be spontaneously pregnant (10 weeks of gestation) and visited our obstetrics and gynecology department. She was followed up with regular checkups, and both mother and child grew well without any problems or the appearance of localized distention or pain in the abdominal wall. At 39 weeks and 0 days of gestation, uterine contractions were observed and the woman was admitted to the hospital for delivery care. The first stage of labor lasted only 13 hours. Although the delivery was delayed due to weak contractions, the uterus was fully dilated after administration of oxytocin. The second stage of labor lasted 28 minutes and the third stage lasted 3 minutes. The baby had an Apgar score of 8 at 1 minute and 9 at 5 minutes, with no abnormalities. Thereafter, there was no localized abdominal wall distention, lower abdominal colic pain, or back pain. One month after delivery, there was no back pain or other symptoms, and a computed tomography scan of the abdomen showed no separation of the rectus abdominis muscle (Fig. 2), so recurrence was not suspected. One year after delivery, the postpartum course was uneventful. These outcomes suggest that retrorectus mesh placement using the enhanced-view totally extraperitoneal approach may allow for safe pregnancy and delivery in selected patients, while preserving abdominal wall function.

## 3. Discussion

In this case, the woman suffered DRA after the birth of her second child. She was initially treated conservatively with exercise therapy to strengthen the transversus abdominis muscle. However, it was not effective, and lower abdominal colic pain and back pain newly appeared. In such cases, surgical intervention is required,^[4]^ and we chose the enhanced-view totally extraperitoneal Rives-Stoppa technique and transversus abdominis release. This approach has the following 3 major advantages: retrorectus mesh reinforcement, flexible port positioning, and adaptation of a standard open surgery method in minimally invasive surgery.^[3]^ The addition of mesh reinforcement also reduces the risk of recurrence compared to simple suture alone. On the other hand, there were concerns that mesh repair might interfere with the next surgery and that chronic pain might occur, as 63% of women reported tearing or traction pain at the repair site during pregnancy after repair surgery.^[5]^ In this case, the transverse diameter of the hernia defect was particularly long, and the elasticity of the abdominal wall was considered to be more limited.^[6]^ Therefore, we explained to the woman that pregnancy after repair was risky. The woman and fetus were carefully monitored throughout the pregnancy. There was no pain, as previously reported, and no obvious abnormalities in fetal development. One possible explanation is that the mesh was not exposed to the abdominal cavity and therefore did not come into direct contact with the uterus. Another possibility is that the mesh may possess a certain degree of elasticity. There was no delay in the second stage of labor, indicating that the abdominal wall function as a delivery force had been restored and that the elasticity of the abdominal wall was maintained by repairing with a sufficiently large mesh. If a cesarean section had been necessary, we might have considered cutting the mesh. Removal of the mesh was considered time consuming and disruptive to the mother and fetus. Whether to remove the mesh and close the hernia defect or simply close the hernia defect without removing the mesh depends on the general condition of the mother. There are no articles in PubMed (1903 to April 2024) that describe the delivery method after mesh repair or delivery care when cesarean section is required. Treatment must be tailored to the condition of the mother and fetus. A systematic review by Jensen and colleagues reported that recurrence of DRA was observed in 2 of 12 pregnancies and births after mesh repair.^[7]^ Both recurrence cases involved semilunar hernias, which may have resulted from an inadequate mesh size. For hernias larger than 10 cm in transverse diameter, transversus abdominis release can be used to secure space for mesh placement. There are few reports on this surgical method in Japan, and no consensus has been reached in Europe and the United States. Placing mesh underneath is not only cosmetically pleasing but also helpful in reducing the risk of intraperitoneal mesh-related complications, such as adhesion to the uterus during pregnancy. Omentum transposition, tissue flaps, or free peritoneal grafts can prevent the bowel from adhering to the mesh.^[8]^ We chose Ventralight Sepra Technology because it has a carboxymethylcellulose sodium hyaluronate coating on 1 side to prevent peritoneal adhesions.^[9]^

## 4. Conclusion

In the treatment of DRA, placement of a mesh in the retroperitoneal space may enable the next pregnancy. Because postoperative pregnancy and childbirth increase the risk of recurrence, it is important to provide patients with appropriate information and discuss the treatment plan with them.^[10]^

This case report describes a single patient’s course following laparoscopic mesh repair for postpartum DRA. Therefore, the generalizability of the findings is limited. The successful pregnancy and vaginal delivery observed in this case may not apply to all patients undergoing similar procedures. Moreover, as the mesh used was a specific type (Ventralight Sepra Technology), outcomes may differ depending on mesh material or surgical technique. Long-term complications, such as chronic pain or recurrence beyond the postpartum period, were not fully evaluated due to the follow-up duration of a few years after delivery. Further studies involving a larger sample size and extended follow-up periods would be beneficial to determine the broader applicability and safety of mesh repair in women considering future pregnancies.

## Author contributions

**Conceptualization:** Shota Eguchi, Yoshio Nagahisa.

**Data curation:** Shota Eguchi, Kenji Yamaguchi.

**Investigation:** Shota Eguchi, Kenji Yamaguchi, Michio Okabe.

**Methodology:** Michio Okabe.

**Project administration:** Yoshio Nagahisa.

**Supervision:** Kenji Yamaguchi, Michio Okabe, Toshihiko Masui.

**Validation:** Michio Okabe.

**Writing – original draft:** Shota Eguchi.

**Writing – review & editing:** Yoshio Nagahisa, Toshihiko Masui.
